# Supplementary figures and images for: Trajectories of medical care expenditure in the last year of life associated with long-term care utilization in frail older adults: A retrospective cohort study
Source: PLoS One. 2024 May 28;19(5):e0297198. doi: 10.1371/journal.pone.0297198 (PMC11132452; doi:10.1371/journal.pone.0297198)

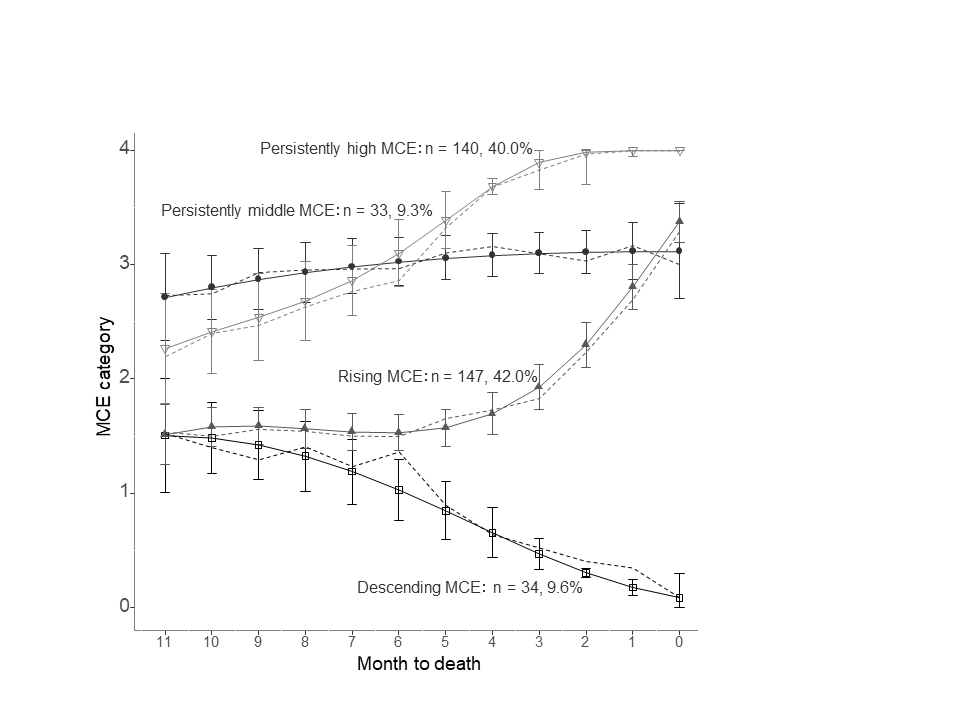

Supplement: S1 Fig — (TIF) [file pone.0297198.s001.tif]
