# Supplementary material for: Trajectories of medical care expenditure in the last year of life associated with long-term care utilization in frail older adults: A retrospective cohort study
Source: PLoS One. 2024 May 28;19(5):e0297198. doi: 10.1371/journal.pone.0297198 (PMC11132452; doi:10.1371/journal.pone.0297198)
Supplement: Attachment — Submitted filename: 2023-1110_Point-by-point_response_to_reviewers.docx [file pone.0297198.s002.docx]

**Point-by-point responses to the reviewer’s comments**

We appreciate the reviewer’s helpful suggestions. Our point-by-point responses to the reviewer’s comments are provided below. In addition to the changes suggested by the reviewer, we have revised some other sentences at various points to improve their flow and comprehensibility and reduce redundancy. The newly added/revised text has been highlighted yellow in the revised manuscript.

***Reviewer 1*** *1) Long-term care is understood differently depending on country context. Suggest providing a few sentences at the Introduction section to set the stage (instead of leaving it till later within the Methods section)*

**Response**

According to your suggestion, we have added a few sentences to the Introduction to set the stage for non-Japanese readers to understand the context of long-term care in Japan (L100-L107).

*2) The first paragraph talks about frailty but then frailty barely mentioned in the remaining article. Even in Table 1, there is no capture of clinical data with respect to frailty (e.g. function, frailty scale, comorbidity severity, cognition, etc). In my opinion, elderly age and presence of a chronic disease is not sufficient to characterize frailty. Suggest rewriting the first paragraph to focus on the significance of studying medical care expenditure in EOL among elderly adults and linking it subsequently to why there is a eventual hypothesis that LTC is associated with different trajectories of care expenditure.*

*The alternative is to include more clinical data/indicators for frailty, of which perhaps what would be also of interest would be frailty predictors for medical care expenditure.*

**Response**

As you suggested, we have rewritten the first paragraph to focus on the significance of studying medical care expenditure and long-term care utilization among older adults at the end of life(L73–L85).

*3) It is interesting that within the Minimal MCE group, the total MCE in last year of life is 0 (0-0). Numbers are also not small (56/405 of cohort). Is this truly zero utilization of medical care? Or is this due to missing data (e.g. patient was recruited into the cohort but left the healthcare coverage area? Some patients did not file insurance claims?) If this is truly due to zero utilization, the authors could provide a statement to declare the comprehensive coverage of the primary outcome measure (healthcare insurance claims). Alternative is to consider a sensitivity analysis of trajectories excluding those patients with zero MCE or imputing their MCE.*

**Response**

Decedents whose total MCE in the last year of life was zero were followed up until their death, meaning the zero MCE was not due to missing data. However, we could not distinguish decedents who did not use medical care genuinely from those who used publicly funded medical care or required public assistance during the follow-up period. We have mentioned this point in the limitations. We assumed that decedents who used publicly funded medical care or required public assistance had not used medical care for more than two months immediately prior to death. Therefore, as you suggested, we have added a sensitivity analysis to estimate the trajectory of medical care expenditure for this group (L200-L203) (L353-L361).

*- Moreover, very few patients (1 per chronic disease type) within these 56 had a known chronic disease. Hence, on deterioration (prior to death), one would expect MCE of some form for further investigations?*

**Response**

As you have clearly noted, for each chronic disease type, only one decedent in the minimal MCE group had a chronic disease. These individuals may have died suddenly without medical care. We have added this explanation to the Discussion (L419–L421), as you suggested.

*- I note that this has been subsequently mentioned in the discussion portion (about th**e lacking of continuous records of medical care utilization in the claim dataset). This missingness may need to be handled or at least written clearly as a limitation.*

**Response**

As you suggested, we have clarified the lack of continuous records of medical care utilization in the claims dataset in the Limitations (L429–L443).

*4) Suggest to reword as associations instead of causations. E.g. “home-based long-term care utilization was associated with increased medical care expenditure in the descending medical care expenditure group. Also I believe the testing of association between long-term care and MCE within group-based trajectory modelling is an incidence rate ratio which should be mentioned?*

**Respons**e

Thank you for your suggestion. We have reworded the explanation as association to avoid causation (L402–L405).

*5) The paragraphs within discussion describing the possible associations with LTC and MCE trajectories are difficult to understand and need to be revised.*

*- For example, for those with rising/persistently high MCE trajectories, a lower incidence rate ratio of MCE is associated with use of facility-based long-term care. The hypothesized reason behind this association should be clearly spelled out. (could it be because facility-based LTC provide some degree of medical/nursing care that reduces the need for hospital MCE?)*

**Respons**e

Thank you for your helpful suggestion. We have added the explanation to the Discussion section (L396–L401).

*- On another note, those with descending MCE trajectories, a higher incidence rate ratio of MCE is associated with use of home-based LTC. What is the hypothesized reason behind this association?*

**Response**

Thank you for the question. Older adults at the end of life need palliative care, including medication, fluid replacement, or tube feeding, to relieve suffering. The patients receiving home-based long-term care might also access home-based medical care through visits by physicians or nurses. We have added this point to the Discussion (L405–L407).

*6) Under limitations, it is mentioned that ICD-10 did not fully assess chronic disease. However, a common methodology is to compute comorbidity severity (either charlson or elixhauser) via ICD-10 codes. This could be considered as a surrogate measure of comorbidity severity since the authors have access to ICD codes*

**Respons**e

We appreciate your suggestion. Our previous description of this limitation was insufficient. We have revised this limitation for clarity (L446–L449).

*7) The fourth limitation is unclear to me. May need to be rephrased.*

**Response**

We think this limitation is not important and have deleted it.

*8) Under conclusion, the first conclusion that there are heterogenous MCE trajectories is quite clear. The subsequent sentences are unclear and I am not sure what the authors are recommending. Are you suggesting that improving on long-term care utilization may attenuate high MCE?*

**Response**

As you suggested, we have revised the conclusion to clarify the recommendation (L462–L464).
